# Metagenomic Sequencing Reveals the Viral Diversity of Bactrian Camels in China

**DOI:** 10.3390/microorganisms13112589

**Published:** 2025-11-13

**Authors:** Jun Li, Ling Hou, Yuhang Liu, Yue Sun, Yong Li, Biao He, Changchun Tu, Xuezhang Zhou

**Affiliations:** 1 Key Laboratory of the Ministry of Education for the Conservation and Utilization of Special Biological Resources of Western China, Ningxia University, Yinchuan 750021, China; lijunamy@126.com (J.L.); hlbelief@163.com (L.H.); liyong7732@nxu.edu.cn (Y.L.); 2 College of Life Science and Technology, Ningxia Polytechnic University, Yinchuan 750021, China; 3 Institute of Basic Medical Science, Ningxia Medical University, Yinchuan 750004, China; 4 Changchun Veterinary Research Institute, Chinese Academy of Agricultural Sciences, Changchun 130122, China; lyh2925020132@163.com (Y.L.); sy3126999@163.com (Y.S.); heb-001001@163.com (B.H.); changchun_tu@hotmail.com (C.T.)

**Keywords:** metagenomics, Bactrian camel, diversity, ecology

## Abstract

The Bactrian camel is a key economic livestock species in China and around the world. It yields meat and milk (high-quality functional foods), and the milk reports health benefits. Dromedary camels, as intermediate hosts of MERS-CoV, have garnered significant public health attention. In contrast, viral surveillance in Bactrian camels from the same genus as dromedaries has received limited attention, with only sporadic or regionally confined reports available. Systematic investigations into the virome of viral species, viral diversity, and novel viruses in Bactrian camels are lacking. In this study, swabs were collected from 701 Bactrian camels in China. Through metagenomics, 3262 viral contigs were classified into 16 viral phyla, 29 viral families, and an unclassified group. The different landforms were found to influence viral diversity and composition in Bactrian camels, with mountainous area exerting the greatest impact. The viral composition significantly differed between captive and free-ranging camels. The study identified at least 12 viruses with zoonotic potential, and phylogenetic analysis indicated cross-species transmission in some of them. Additionally, picornavirus, circular Rep-encoding single-stranded (CRESS) DNA virus, and polyomavirus from Bactrian camels may represent novel species or genotypes. To summarize, in this study, we described the baseline virome profile of Chinese Bactrian camels, investigated the ecological factors influencing the viral distribution of Bactrian camels, identified key potential viral risks, and provided a scientific basis for the prevention, control, and early warning of critical viral diseases in Bactrian camels from China.

## 1. Introduction

Camels are among the most unique mammals and thrive under conditions of extreme temperatures, scarce vegetation, and very limited availability of food and water. Camels are mostly classified into two species, Dromedary camels (*Camelus dromedarius*) and Bactrian camels (*Camelus bactrianus*). Bactrian camels, distinguished by two humps, include *Camelus bactrianus*, which mainly inhabits northwestern China, and *Camelus ferus*, which lives in Central Asia [1]. China is one of the major locations for the breeding of Bactrian camels. The domestic Bactrian camel population in China reached 580,000 individuals in 2023, distributed across Xinjiang, Inner Mongolia, Gansu, Qinghai, and Ningxia (https://www.stats.gov.cn/sj/ndsj/ (accessed on 5 January 2023)). They are used in transport, sports, and also yield meat and milk [2,3]. Therefore, they contribute to increasing the economy and food security of the country [4]. Owing to its economic benefits and expansion of the farming scale, active surveillance and prevention of diseases are crucial for the sustainable development of camel populations in China.

With advancements in research on zoonotic diseases, studies have reported that dromedary camels serve as significant reservoirs and transmission sources for multiple highly pathogenic, rapidly spreading viruses (such as MERS-CoV), posing substantial public health risks [5,6,7]. In contrast, current reports of infections in Bactrian camels, which are congeneric with Dromedary, are limited to regional occurrences of Mamastroviruses, hepatitis E virus and foot-and-mouth disease, etc. [8,9,10,11]. However, systematic investigations of pathogenic viruses in Chinese Bactrian camels are lacking. This highlights the urgent need to reveal the baseline of viral pathogen and explore the ecological factors affecting its distribution. This study provides foundation for the targeted control of viral diseases in Bactrian camels and the evaluation of public health risks.

The development of viral metagenomics has considerably increased our ability to assess viral diversity and has provided critical insights for pathogen surveillance [12,13]. Global ocean RNA sequences have been analyzed to expand RNA virus catalogs and their taxonomy [14]. Studies on the viromes of rodents, bats, wild birds, ticks and fur animals have revealed multiple viruses with zoonotic potential, including SARS-CoV-2, influenza A virus strains (H1N2, H5N6, and H6N2), HKU5-like coronaviruses, and avian influenza A virus H9N2 [15,16,17,18,19,20]. With respect to viral metagenomic sequencing in camels, studies have reported viral metagenomic analyses of nasopharyngeal swabs, fecal samples, and ticks collected from dromedary camels. These studies have identified sequences related to mammalian viruses from several viral families, including *Hepeviridae*, *Coronaviridae*, *Nairoviridae*, *Paramyxoviridae*, *Polyomaviridae*, *Papillomaviridae*, *Astroviridae*, *Picornaviridae* and *Genomoviridae* [21]. A high diversity of contigs from the *Circoviridae* and *Picobirnaviridae* families was observed in dromedary camel fecal samples [22]. Notably, some viruses identified in NP swab samples, such as Crimean Congo hemorrhagic fever virus (CCHFV), PIV3, PIV4, and camel bocavirus 3, are potentially novel strains. The human disease-associated viruses and three novel viruses detected in ticks collected from Dromedary camels include CCHFV, Tamdy virus (*Nairoviridae*), Guertu virus (family *Phenuiviridae*), and Iftin tick virus, Bangali torovirus, Bole tick virus 4 [23]. In stark contrast, the current viral metagenomics research on Bactrian camels is limited to a single study by Pan et al. on 17 camels from Wuwei, Gansu Province, which represents a small sample size and restricted area [24]. Our study represents the first in-depth and systematic analysis of viral diversity in Chinese camel populations using this methodology.

In this study, we collected swab samples from Chinese Bactrian camels and investigated various ecological factors associated with their breeding environment. Viral metagenomic sequencing was subsequently conducted to analyze the viral composition and diversity in Bactrian camels. We obtained the baseline metagenomic profile of Chinese Bactrian camels and investigated the ecological factors influencing viral distribution in Bactrian camels.

## 2. Materials and Methods

### 2.1. Swab Sample Collection from Chinese Bactrian Camels

Nasal, pharyngeal, or anal swabs were collected by professional veterinary staff with the consent of the Bactrian camel owners and herders, and none of the surveyed Bactrian camels had any previous disease. Based on the sampling location, the samples were placed in the sampling box, and the sampling location, time, type, and quantity were recorded (Table S1). Each sample was frozen in liquid nitrogen immediately after collection. All the samples were transported to the laboratory with dry ice and stored at −80 °C until use.

### 2.2. Sample Pretreatment and High-Throughput Sequencing

A total of 701 Bactrian camel samples from 22 locations were processed, resulting in 22 separate sample pools. Each pool consisted of nasal, pharyngeal, and anal swabs collected from 20 to 61 Bactrian camels at a single location (see Table S1 for the number of Bactrian camels per location). Each pool was labeled according to the sampling location (e.g., ‘GCSW’). In brief, appropriate volumes of nasal, pharyngeal, and anal swab samples from the same sampling locations were mixed in a 1.5 mL microcentrifuge tube. The mixture was subsequently centrifuged at 12,000× *g* for 10 min at 4 °C (Eppendorf, Hamburg, Germany). The supernatant was subsequently collected and filtered through a 0.22 μm membrane to obtain the filtrate. Next, 260 μL of filtrate was directly used for total RNA extraction using the TRIzol reagent (Invitrogen, Carlsbad, CA, USA). The viral DNA was extracted from 500 µL of the filtrate using the HiPure Swab DNA Kit (Magen, Guangzhou, China) and isothermally amplified using an Illustra GenomiPhi V2 DNA amplification kit (GE, Fairfield, CT, USA) following the manufacturer’s instructions. The quality of the amplification products was measured using a NanoDrop One spectrophotometer (Thermo Scientific, Waltham, MA, USA), and 1.0% agarose gel electrophoresis was performed. All amplified products were stored at −80 °C until the library was constructed. The consumables used in sample processing were all RNase-free, and all operations were performed in a biosafety cabinet. 

The RNA-seq libraries were constructed using the NEBNext Ultra Directional RNA Library Prep Kit (NEB, Ipswich, MA, USA) following the manufacturer’s protocol. DNA libraries were constructed using the ALFA-SEQ DNA Library Prep Kit (Finorop, Beijing, China). The quantity and quality of the libraries were assessed using a Qubit^®^ dsDNA HS Assay Kit (Life Technologies, Grand Island, NY, USA) and an Agilent 4200 system (Agilent Technologies, Santa Clara, CA, USA). The paired-end libraries were sequenced at 2 × 150 bp on the Illumina NovaSeq 6000 platform (Illumina, San Diego, CA, USA). Each pool yielded at least 6 Gb of data.

### 2.3. Viromic Annotation

The quality of the raw reads was evaluated via the fastp software (v. 0.20.0), which removed low-quality reads, low-complexity reads, and undetermined bases. High-quality sequencing data, referred to as clean data, were generated. The clean reads were subsequently mapped against the host sequence database (*Camelus bactrianus*, GenBank Accession number GCA_000767855.1) to remove host genome sequences using Bowtie2 (2.3.1), followed by rapid metagenomic classification of bacterial, archaeal, and fungal genomes using Kraken2 (version 2.0.9) and a custom RefSeq-based database (https://www.ncbi.nlm.nih.gov/refseq/ (accessed between 1 January 2024 and 30 December 2024)),. The remaining reads of RNA and DNA viruses were de novo assembled into contigs using MEGAHIT (v1.1.3). These contigs were annotated using blastn and diamond blastx searches (e-value ≤ 1 × 10^5^) against the eukaryotic viral reference database (EVRD)-nt/aa version 3.0 [25]. Finally, each alternative sequence was compared to a nonredundant nt/nr database for online validation by BLASTn/x (https://blast.ncbi.nlm.nih.gov/Blast.cgi (accessed between 1 January 2024 and 30 December 2024)). The sequences were considered to be virus-like contigs (VLSs) if they had the best hits with amino acid or nucleotide sequences of the viruses. The viral sequences were classified taxonomically at 90% and 50% similarity levels. Viral sequences with ≥90% similarity to known sequences were classified at the genus level, whereas those with ≥50% similarity were classified at the family level. Thus, the final VLSs were condensed using CD-HIT v.4.8.1 at 99% similarity over 80% coverage and denoted as the Bc-Virome. The overall diversity of the Bc-Virome was assessed at the species (ANI90) and subgenus (AAI90) levels. The aa sequences of the Bc-Virome were predicted by Prodigal. Bc-Virome nt and aa sequences were clustered at the respective similarities over 90% and 80% coverage (for ANI90 and AAI90) using MMseqs2 (version 7e2840992948ee89dcc336522dc98a74fe0adf00) with the coverage mode of 0.

### 2.4. The Hallmark Genes of the Virus Sequence

Unless otherwise specified, all the ecological and statistical analyses were performed using the viral hallmark genes (VHGs) dataset of the Bc-Virome. VHGs are broadly conserved among different groups of viruses. RdRp is a super-VHG that unites almost all RNA viruses. The RdRp domains of the Bc-Virome were identified by scanning the Bc-Virome aa sequences using hmmsearch against the RdRp profiles of iVirus and RdRp-scan. The major capsid protein (MCP) is another super-VHG that is encoded by almost all DNA viruses. The curated MCP dataset was built on the UniProt database. The MCP domains of the Bc-Virome were identified by querying the related sequences against the MCP dataset using DIAMOND BLASTP version 2.1.8. All the MCP candidates were validated by checking the conserved motifs. Bc-Virome were classified as full-length if they aligned with more than 80% of the average alignment length. All RdRp and MCP sequences of the Bc-Virome were pooled and condensed using cd-hit at 95% nt similarity over 80% coverage.

### 2.5. Viral Abundance Quantification and Ecological Analysis

For the viral abundance analyses, the unclassified reads from each pool were mapped against Bc-Virome VHG sequences using Bowtie2 with end-to-end sensitivity. The mapped reads were counted using SAMtools v.1.10. Reads with a count of ≤10 were removed to minimize index hopping. The read count of each VHG within a vcANI90 was summed to represent the read count of the vcANI90. The relative abundance of each vcANI90 of VHGs was calculated by dividing the million unclassified reads by the read count of the vcANI90 (i.e., reads per million mapped reads, RPM); RPM ≤ 1 was removed to further minimize index hopping. Species accumulation curves for all the libraries were generated using the vegan package, applying the random method with 10,000 permutations. Within-library diversity, measured using the Shannon and Simpson diversity indices for each pool, was deduced using the ‘diversity’ function of the vegan package. The differences in alpha diversity among the different rearing modes, ages, and landforms were compared using the Wilcoxon rank sum test. Bray–Curtis dissimilarity matrices between pools were subsequently computed using the vegdist function from the vegan package. Beta diversity patterns were ordinated via non-metric multidimensional scaling (NMDS, metaMDS function, vegan v2.6–4) to visualize dissimilarities in viral community composition among the different groups. Significant differences in the composition of viruses were determined by permutational multivariate analysis of variance (PERMANOVA) with 9999 permutations using the vegan package. Viral genomic structural types were visualized using proportional treemaps.

### 2.6. Molecular Detection and Phylogenetic Analysis

To infer viral evolutionary relationships, phylogenetic analysis was performed for each viral family using VHG representatives of the Bc-Virome. The reference sequences and associated host metadata were obtained from NCBI, ICTV, and the Virus–host Database. The sequences were aligned using MAFFT v7.520 and trimmed using trimAL v.1.4 to remove columns containing ≥ 67% gaps. An initial maximum-likelihood tree was reconstructed using IQ-TREE v.1.6.12 with 1000 ultrafast bootstrap values, and the best-fit model was automatically determined.

To validate the Bc-Virome, we performed nested RT-PCR and conventional PCR assays to specifically detect RNA and DNA viruses, respectively. The primers were designed with Primer Premier 5 to target the contigs of the detected virus (Table S2). Viral RNA and DNA were extracted using a HiPure Viral RNA/DNA Kit (Magen, Guangzhou, China), and RNA was reverse-transcribed using a PrimeScript^™^ II 1st Strand cDNA Synthesis Kit (TaKaRa, Dalian, China) following the manufacturer’s protocol. PCR amplification was conducted with 2× Rapid Taq Master Mix (Vazyme, Nanjing, China) following the manufacturer’s protocol. All reactions included purified water as a negative control.

## 3. Results

### 3.1. Subsection

#### 3.1.1. Overview of the Bc-Virome

**Figure 1:**
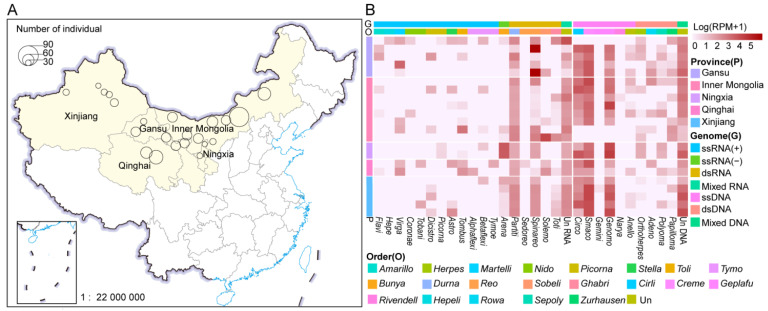
Overview of the samples and viromes of Bactrian camels. (**A**). Geographical sampling locations are indicated by light yellow shading on the map of China. The pie charts on the map show the sample types and numbers (*n* = 701), with each color representing a sample type. The base layer of this modified map was obtained from the National Earth System Science Data Center, National Science & Technology Infrastructure of China (http://www.geodata.cn (accessed on 4 May 2025)); (**B**). The diversity and richness of viruses associated with vertebrates and invertebrates in Bactrian camels were assessed. The viruses identified from 29 viral families that belong to seven viral types (positive-sense ssRNA virus, negative-sense ssRNA virus, dsRNA virus, mixed RNA virus, ssDNA virus, dsDNA virus, and mixed DNA virus) are shown. The relative abundance of viruses in each library was calculated and normalized to the number of mapped reads per million (RPM) total reads. The read abundances of different viral families in each library are shown from white to dull red, indicating an increasing tendency. The colors on the left of the heatmap represent different sampling locations. Viral family, order, and phylum names were trimmed by removing common suffixes. Relative abundances are expressed in RPM. Viral names ending with ‘un’ represent unclassified viruses that could not be assigned to any known viral family.

From February 2023 to November 2024, a total of 2101 swabs were collected from 701 individual asymptomatic Bactrian camels in five provinces of China, including Xinjiang (*n* = 147), Inner Mongolia (*n* = 277), Gansu (*n* = 143), Qinghai (*n* = 80), and Ningxia (*n* = 54), including 701 nasal swabs, 701 pharyngeal swabs, and 699 anal swabs (Figure 1A). Among these samples, anal swab samples from two Bactrian camels in Urumqi County, Xinjiang, were unfortunately lost. Following the library construction method described in Section 2.2, 22 sample pools from the swabs of Bactrian camels were, respectively, used to perform virome analysis via the MTT and MDA approaches, resulting in the construction of 44 libraries (22 DNA libraries and 22 RNA libraries) [26]. Ultimately, the MTT and MDA approaches yielded the data (Table S3), which were subsequently used for downstream bioinformatics analyses. A total of 3262 viral contigs were obtained. At least 1277 complete genomes or those encompassing the complete coding region have been identified. The Bc-Virome covers eight RNA and eight DNA viral phyla. The viral contigs were assigned to 29 viral families. Analysis of viral abundance across five provinces revealed the lowest value in Xinjiang and the highest in Qinghai (Figure 1B).

The species accumulation curves generated via vcANI90 from different libraries revealed that as the number of samples increased, the curve plateaued, indicating that the number of libraries collected in this study was sufficient to meet the analytical requirements (Figure S1A).

The Bc-Virome has a variety of genome types, including ssRNA(+), ssRNA(−), dsRNA, ssDNA, and dsDNA viruses. Owing to the ability of the MDA technique to amplify single-stranded circular DNA molecules, circular Rep-encoding single-stranded (CRESS) DNA viruses were overrepresented in the Bc-Virome, accounting for 66.08% (*n* = 1582) (Figure S1B). We also identified 1277 full-length VHGs of the Bc-Virome, i.e., 86 RNA-dependent RNA polymerase (RdRp) sequences and 1191 MCP sequences. At the subgenus level (i.e., sequences with an average amino acid identity of 90% [AAI90] over 80% coverage), these VHG sequences were grouped into 786 viral clusters (vcAAI90). However, only four vcAAI90s presented >50% positive rates in the paired libraries (Figure S1C). This finding suggested that the viromes of Bactrian camels varied significantly among the libraries.

#### 3.1.2. Ecological Factors Influencing Viral Diversity

**Figure 2:**
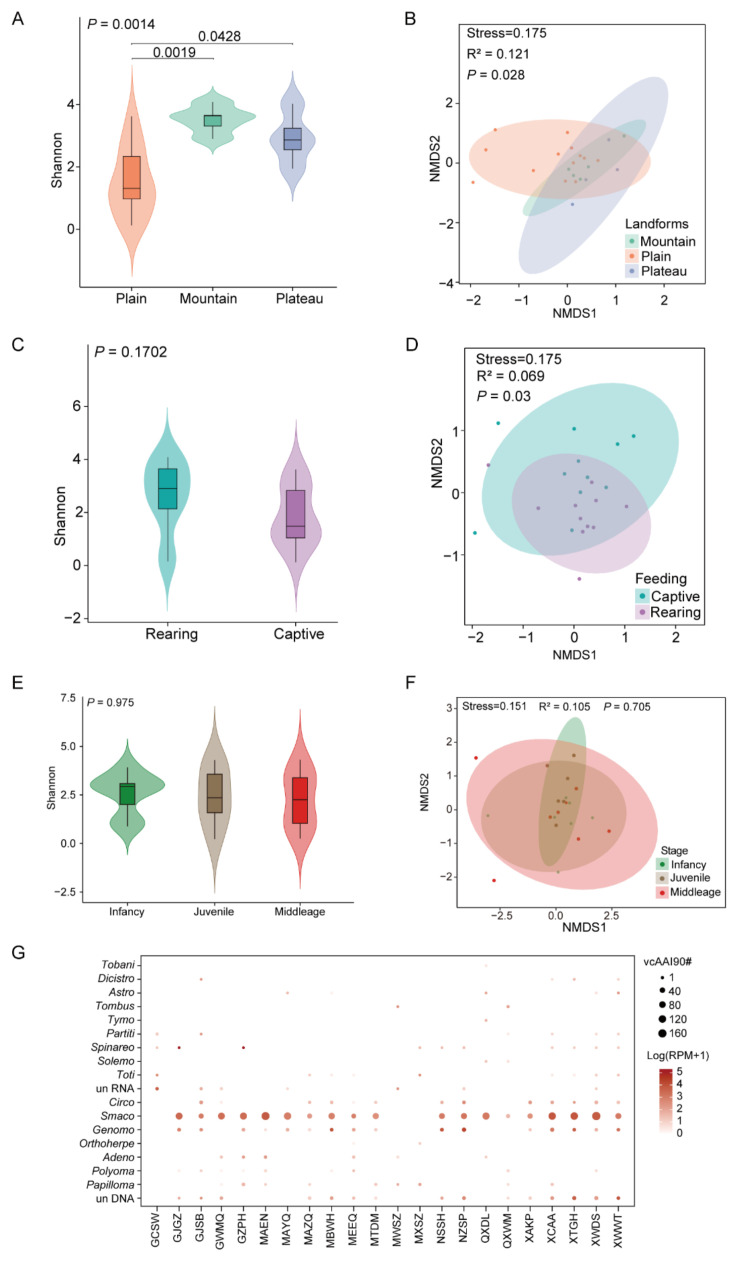
Different factors shaping the virome composition of Bactrian camels. (**A**,**C**,**E**) Violin plots showing the α diversity of eukaryotic viruses under different landforms, rearing models, and ages. Shannon α diversity indices, computed based on the relative abundance of species (Vegan package v2.6–4), were analyzed for intergroup differences via Wilcoxon rank-sum tests with Benjamini-Hochberg correction. (**B**,**D**,**F**) Non-metric multidimensional scaling (NMDS) analysis was performed to show the variations in viral composition under different landforms, rearing models, and ages. In the NMDS plots, the circles represent the 95% normal probability ellipse. (**G**) Viral family distribution across sampling sites is shown. Bubble sizes represent relative quantities of vcAAI90 clusters (90% amino acid identity) for each viral family detected at each sampling location.

We investigated the viral diversity and composition of different landforms, ages, and rearing models. The number and percent of animals and sample pools across different landforms, breeding modes, and age groups are presented in Table S4. Alpha diversity analysis revealed substantial variability in viral diversity across different landforms (Wilcoxon test, *p* < 0.05). Notably, Bactrian camels in the Mountainous area exhibited the highest viral diversity, followed by those in the plains and plateaus (Figure 2A). Further NMDS analysis revealed significant differences in the viral composition of Bactrian camels among the three different landforms (Figure 2B). In terms of the rearing model, the viral diversity did not differ significantly between captive and free-ranging individuals (Figure 2C). In comparison, significant variations in β diversity were found between rearing modes, indicating distinct overall viral compositions between captive and free-ranging (R^2^ = 0.069, *p* = 0.03). We ruled out the possibility that the differences between rearing modes were driven by data dispersion within each group through PERMANOVA (Figure 2D). Additionally, no significant differences in viral richness or composition were found among the different age groups of Bactrian camels, suggesting limited age-associated effects on viral diversity and community structure (Figure 2E,F).

Viral species richness was significantly lower in Bactrian camels from Inner Mongolia than in those from Xinjiang, Gansu, Qinghai, and Ningxia. Following vcAAI90-based dereplication, DNA viruses showed a broad distribution across sample pools, with complete absence only in Jinchang, Gansu. RNA viruses were less abundant across most sample pools. In contrast, samples from Xinjiang and Qinghai presented greater RNA viral diversity, predominantly comprising the families *Astroviridae*, *Spinareoviridae*, and *Tobaniviridae*. Most DNA viral contigs from the different sampling locations were classified CRESS-DNA viruses. *Smacoviridae* was the most abundant family, followed by *Genomoviridae* and *Circoviridae*. The high prevalence of CRESS DNA viruses was correlated with significant genetic divergence, highlighting that there are knowledge gaps in terms of their replicative mechanisms and zoonotic risk assessment (Figure 2G).

#### 3.1.3. Phylogenetic Analysis of Key Viral Sequences

After rigorous screening, we identified at least 12 viruses with zoonotic potential belonging to seven viral families and nine genera. These viruses can cause diseases such as diarrhea, respiratory symptoms, hepatitis and cervical cancer in animals or humans. These viruses include but are not limited to Mammalian orthoreovirus 1 (*Spinareoviridae*, dsRNA), Parainfluenza virus 5 (*Paramyxoviridae*, ssRNA(−)), Bovine coronavirus (*Coronaviridae*, ssRNA(+)), Hepatitis E virus (*Hepeviridae*, *ssRNA*(+)), Dromedary astrovirus and Porcine astrovirus (*Astroviridae*, ssRNA(+)), Bovine torovirus and Goat torovirus (*Tobaniviridae*, ssRNA(+)), and human papillomaviruses (*Papillomaviridae*, dsDNA). Owing to the unexpectedly high genetic variability of CRESS DNA viruses in our dataset. We conducted detailed phylogenetic analyses of these viruses.

##### Double-Stranded RNA Viruses

**Figure 3:**
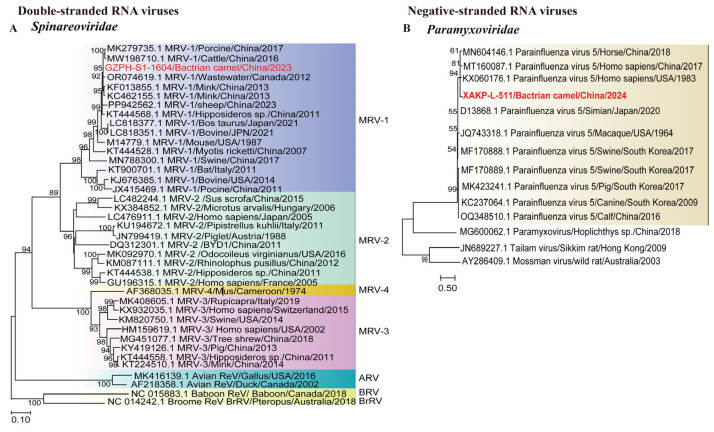
Phylogeny of mammal-associated Double-stranded and Negative-sense RNA viruses. The phylogenetic trees show Double-stranded and Negative-sense RNA viruses associated with mammals. (**A**): Bayesian inference tree established based on the S1 sequence of *Spinareoviridae*; (**B**): Bayesian inference tree established based on the partial L nt sequence of *Paramyxoviridae*. Within trees in A and B the viruses found in this study are marked with red font in bold. Each scale bar indicates the amino acid or nucleotide substitutions per site. Different taxonomic clusters are represented by rectangles filled with different colors or Lines in different colors. Taxon names are indicated in light with the corresponding colors. The corresponding host phylogenies are also shown beside the large tree. Bootstrap values >50 is indicated on the trees and the sizes of the dots on the nodes correspond to the bootstrap values.

We obtained 18 *Spinareoviridae* sequences, with lengths ranging from 505 to 4103 nucleotides. One 1604 bp contig was the S1 sequence of a dsRNA virus. Phylogenetic analysis of the S1 sequence revealed that the contig clusters were most closely related to porcine-derived MRV-1, which presented 99.03% S1 sequence identity. (Figure 3A). This discovery improved our understanding of the host range of *Spinareoviridae*, indicating that cross-species transmission may have occurred.

##### Negative-Sense RNA Viruses

One ssRNA(−) virus identified here belongs to *Paramyxoviridae*. The 511 bp contig from a Bactrian camel sample clustered with parainfluenza virus 5 (PIV5), showing 99.80% partial L nucleotide (nt) sequence identity, indicating close genetic relationships (Figure 3B). PIV5 is classified within seven other viruses in the genus *Orthorubulavirus*, one of two genera within Rubulavirinae. PIV5 is globally distributed and is associated with respiratory disease in canines, cattle, swine, and lesser pandas [27,28,29,30]. This finding highlights the need to assess the zoonotic risks posed by parainfluenza viruses in camel populations.

##### Positive-Sense RNA Viruses

Our results revealed the presence of eight ssRNA(+) viruses from five families, including one *Coronaviridae* virus, one *Hepeviridae* virus, three *Astroviridae* viruses, one *Picornavirdae* virus, and two *Tobaniviridae* viruses.

**Figure 4:**
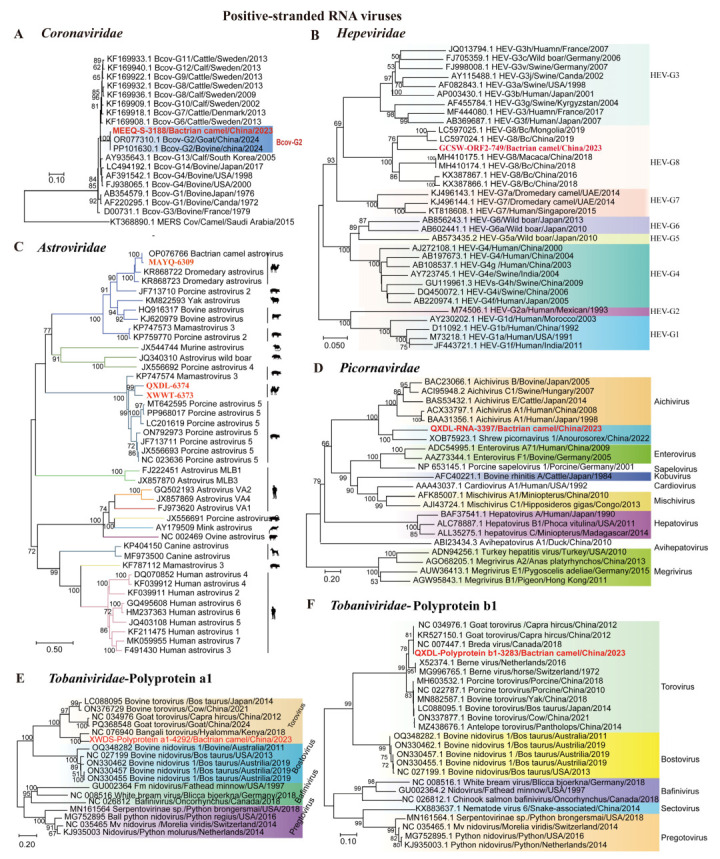
Phylogeny of mammal-associated positive-sense RNA viruses. The phylogenetic trees show positive-sense RNA viruses associated with mammals. (**A**–**F**) Bayesian inference tree established based on the BCoV S gene sequences of *Coronaviridae*, partial ORF2 sequences of *Hepeviridae*, complete genomes of *Astroviridae*, partial Cap protein of *Picornavirdae*, partial ORF1a gene nucleic acid sequences of *Tobaniviridae*, and ORF1b of *Tobaniviridae*. Within trees in (**A**–**F**) the viruses found in this study are marked with red font in bold. Each scale bar indicates the amino acid or nucleotide substitutions per site. Different taxonomic clusters are represented by rectangles filled with different colors or lines in different colors. Taxon names are indicated in light with the corresponding colors. The corresponding host phylogenies are also shown beside the big tree. Bootstrap value >50 are indicated on the trees and the sizes of the dots on the nodes correspond to the bootstrap values.

A nearly complete S sequence (3188 bp) of coronavirus was obtained from Bactrian camel samples in Ordos, Inner Mongolia, using nested RT-PCR. The phylogenetic analysis revealed that the coronavirus S sequence was located in the same cluster as the bovine coronavirus (BcoV) strains from goats and bovines, belonging to BcoV G2 (Figure 4A). This finding is consistent with BcoVs previously identified in ruminants, as reported in another study [31]. Furthermore, BcoV was detected in 31.6% (3/81) of the samples from the viral sequence library by RT-PCR.

We identified one *Hepeviridae* contig of 749 bp from Bactrian camel sample. Phylogenetic analysis revealed that the contig was clustered in a clade with hepatitis E virus genotype 8 (HEV-8), showing 85.39–85.66% identity with HEV-8 from Bactrian camels [32] (Figure 4B). These results indicate the presence of genetically diverse HEV-8 strains in the Bactrian camel population, emphasizing the need for further investigation into their genetic diversity. Using RT-PCR, we detected 15 positive samples (16.3%, 15/92) among the 92 samples collected.

In total, 12 high-quality contigs were identified as *Astroviridae* across all sample pools. Among these contigs, three nearly complete astrovirus (AstV) genomes were obtained from Alxa Right Banner (Inner Mongolia), Ürümqi (Xinjiang), and Delingha (Qinghai), with lengths of 6373 bp, 6374 bp, and 6309 bp, respectively. Phylogenetic analysis revealed that AstVs detected in Bactrian camels were not monophyletic. These astroviruses were grouped into two clades in the genus *Mamastrovirus*. One clade in which MAYQ-6309 from the Bactrian camel clustered with BcAstVs in Inner Mongolia (China) shared 82.95–92.18% whole-genome identity. Additionally, QXDL-6374 and XWWT-6373 constituted a well-supported subclade (bootstrap ≥ 90%) nested within the porcine astrovirus 5 lineages, sharing 75.51–89.09% whole-genome identity (Figure 4C). These results indicated that cross-species transmission may have occurred. Furthermore, RT-PCR detection of AstVs in 75 swab samples revealed five positives, corresponding to a positive rate of 10.7% (5/75).

We also detected 4 *Picornaviridae* contigs, including one partial Cap contig (3397 bp) from the Bactrian camel. Phylogenetic analysis of P1 sequences showed that QXDL-RNA-397 formed a clade with Yunnan shrew picornavirus 1 from *Anourosorex*, which shared 42.38% amino acid (aa) identity (Figure 4D). Notably, this sequence did not cluster with viruses from other genera (*Aichivirus*, *Enterovirus*, *Sapelovirus*, *Kobuvirus*, and *Cardiovirus*), supporting its potential discovery of novel strains or species [33].

We assembled 13 *Tobaniviridae* sequences, with two partial ORF1a and ORF1b nucleotide sequences (4292 bp and 3283 bp). Phylogenetic analysis of the ORF1a and ORF1b sequences revealed that both sequences belong to the genus *Torovirus*, forming a distinct clade separate from other related genera such as *Bostovirus* and *Bafinivirus*. QXDL-Polyprotein a1-4292 formed a clade with Bangali torovirus from Hyalomma rufipes, exhibiting 85.62% nucleic acid identity (Figure 4E), while QXDL-polyprotein b1-3283 clustered with Breda and goat torovirus from calves and goats, sharing 92.33–92.75% nucleic acid identity (Figure 4F). This broad host range suggests extensive and complex transmission routes for *Tobaniviridae* viruses, which may facilitate viral evolution and genetic diversification, making these viruses potential public health concerns.

##### Double-Stranded DNA Viruses

The 14 dsDNA viruses identified here fell into four groups: *Adenoviridae*, *Orthoherpesviridae*, *Polyomaviridae*, and *Papillomaviridae*.

**Figure 5:**
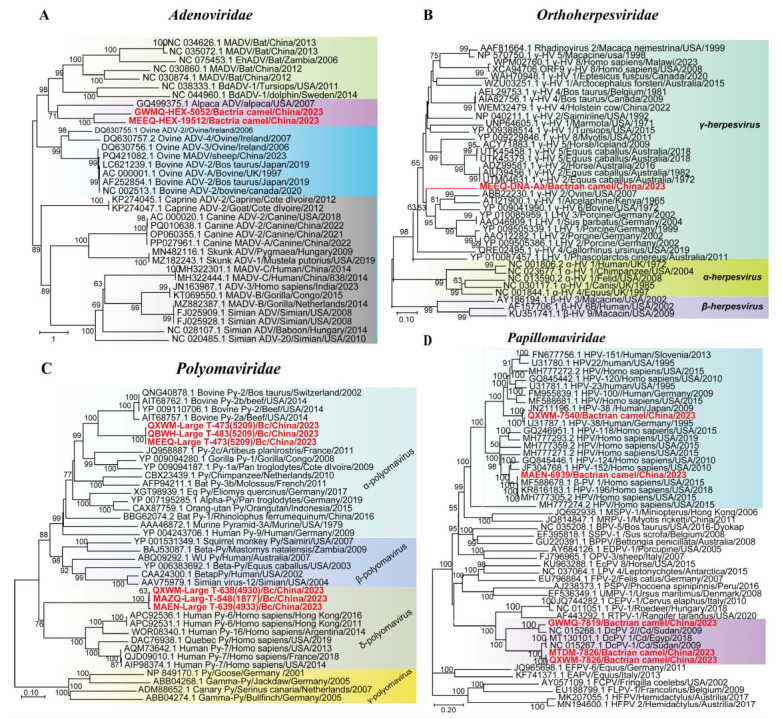
Phylogeny of mammal-associated DNA viruses. The phylogenetic trees show DNA viruses associated with mammals. (**A**–**D**) Bayesian inference tree established on the basisi of the partial Hexon gene sequence of *Adenoviridae*, the D-pol amino acid sequence of *Orthoherpesviridae*, the complete genomes of *Papillomaviridae*, and the Large-T-antigen aa sequences of *Polyomaviridae*. Within trees in (**A**–**D**) the viruses found in this study are marked with red font in bold. Each scale bar indicates the amino acid or nucleotide substitutions per site. Different taxonomic clusters are represented by rectangles filled with different colors. Taxon names are indicated in light with the corresponding colors. The corresponding host phylogenies are also shown beside the large tree. Bootstrap values >50 are indicated on the trees and the sizes of the dots on the nodes correspond to the bootstrap values.

We detected 7 adenovirus contigs, including two partial Hexon gene adenovirus contigs (1930–1939 bp) from Bactrian camels. Phylogenetic analysis of the Hexon gene revealed that contigs GWMQ-HEX-1930 and MEEQ-HEX-1939 formed a clade. This clade was sister to Alpaca adenovirus, sharing 74.67% nucleotide identity, and was clustered within a major clade predominantly composed of ovine adenoviruses (Figure 5A). These results revealed that two Bactrian camel-derived contigs belong to the genus *Mastadenovirus* and may represent the potential discovery of novel strains or species.

We obtained 18 contigs belonging to the family *Orthoherpesviridae*. Among these, a 1382 bp D-pol sequence from Bactrian camel were subjected to phylogenetic analysis, which classified the virus into the subfamily *Gammaherpesvirinae* (Figure 5B). Phylogenetic analysis of this subfamily revealed a clear pattern of host-specific clustering, with viruses from porcine, ovine, bovine, sea otters, and alcelaphine forming five distinct monophyletic clades. MEEQ-Dpol-1382 clusters within a major clade of gammaherpesvirus lineage but forms a distinct subclade, sharing 60.73–65.00% amino acid identity with its closest relatives. Gammaherpesviruses exhibit a broad host range, infecting certain avian and reptilian species, but mammalian species serve as their primary hosts [33]. These findings highlight the presence of potentially novel or highly divergent strains.

Six polyomaviruses large-T-antigen aa sequences (473–639 aa) from Bactrian camels were assembled. Phylogenetic analysis revealed that three sequences (QXWM-473, QBWH-483, and MEEQ-473) clustered a well-supported clade (99% bootstrap) with bovine polyomavirus 2 from the genus *Epsilonpolyomavirus*, sharing 66.42–66.67% amino acid identity. The other three sequences (QXWM-638, MAZQ-548, and MAEN-639) clustered within a major clade containing human polyomaviruses from the genus *Detapolyomavirus*, exhibiting 41.63% to 43.20% amino acid identity (Figure 5C). According to ICTV guidelines [34], two polyomaviruses belong to the same species if the amino acid sequence identity of the LTAg is greater than 70%. Given that the six polyomavirus sequences from Bactrian camels presented only 41.63–66.67% identity to all recognized polyomaviruses, we propose that they may represent novel viral species found in Bactrian camels.

Five complete/near-complete papillomavirus genomes (6939–7826 bp) from Bactrian camels were identified. Phylogenetic analysis revealed that these sequences formed primarily into two distinct clades corresponding to human papillomaviruses (HPVs) and *Camelus dromedarius* papillomaviruses. QXWM-7540 and MAEN-6939 clustered with HPV type 38 and the human-derived Betapapillomavirus 1, respectively. The other three sequences (GWMQ-7819, QXWM-7826 and MTDM-7826) formed a sister lineage to *Camelus dromedarius* papillomavirus type 1 and 2, sharing 91.81–93.43% nucleotide identity (Figure 5D). These findings reveal a remarkable host diversity of papillomaviruses, with strains related to HPVs in Bactrian camels.

##### CRESS DNA Viruses

**Figure 6:**
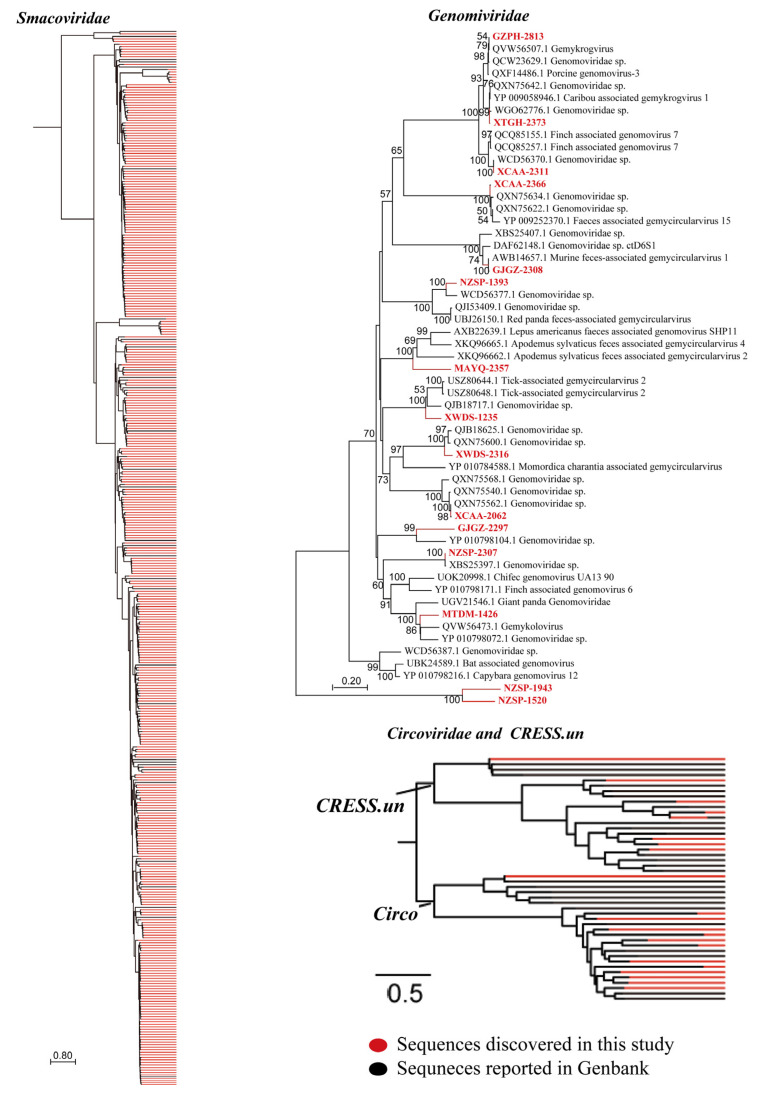
Bayesian inference tree established based on amino acid sequences of the RdRp protein of CRESS-DNA viruses. Each scale bar represents the number of amino acid or nucleotide substitutions per site. The sequences discovered in this study are shown in red, and the known sequences in GenBank are showed in black.

CRESS-DNA viruses are widespread and infect nearly all eukaryotic organisms globally. They are known to induce secondary infections by compromising the immune system of the host, particularly through lymphocyte depletion and dysfunction. Currently, the ICTV classifies these viruses into six families, including *Circoviridae*, *Genomoviridae*, *Geminiviridae*, *Nanoviridae*, *Bacilladnaviridae*, and *Smacoviridae*. In this study, we recovered CAP protein sequences from our metagenomic datasets and detected 13 genomoviruses, 384 smacoviruses, 21 circoviruses, and unclassified CRESS DNA viruses from Bactrian camel samples. Phylogenetic analysis of the CAP amino acid sequences revealed that *Smacoviridae* sequences clustered into 10 distinct clades, and *Genomoviridae*, *Circoviridae* and unclassified viruses each formed distinct phylogenetic branches divergent from reference taxa. Compared with other known smacoviruses, the CAP amino acid sequences of identified smacoviruses presented amino acid identities of 27.1–87.5% (Figure 6). These findings revealed the remarkable evolutionary divergence of CRESS DNA viruses. Currently, within this broad viral group, mammalian pathogenicity of CRESS DNA viruses is associated primarily with the *Circoviridae* family (particularly porcine circoviruses), whereas the clinical significance of other families (e.g., *Smacoviridae*) remains largely unexplored. Therefore, future studies employing animal models and epidemiological investigations are warranted to assess their potential health threats. As an initial step, we randomly detected 64 samples from two libraries for circovirus verification via PCR. Among them, 13 tested positivity, corresponding to a positive rate of 20.3%.

## 4. Discussion

Here, we aimed to construct the baseline virome profile of Bactrian camels in China. Studies on viral carriage in Bactrian camels are limited [34]. An analysis of viral prevalence was conducted using 17 Bactrian camel samples collected from Wuwei, Gansu, along with other herbivorous livestock, and the results revealed that camels presented high CRESS DNA viral loads, particularly those of *Genomoviridae* strains [24]. However, the small sample size of Bactrian camels and the lack of systematic virome characterization limit the generalizability of these findings. Since the nose, pharynx and anus are major sites for viral infection or shedding, we collected 2101 swabs from 701 asymptomatic Bactrian camels in five provinces of China, encompassing all major Bactrian camel farming regions in China. This comprehensive coverage serves as a fundamental prerequisite for systematically investigating viral carriage in Chinese Bactrian camels. We detected 29 viral families and an unclassified group. This profile was largely consistent with the mammalian viral families and diversity identified in nasopharyngeal and fecal samples from dromedary camels, including families such as *Coronaviridae* and *Hepeviridae*. However, viruses such as MERS-CoV, CCHFV, and related tick-borne viruses were not detected in Bactrian camels.

Some studies have revealed that geographical factors such as climate, landforms, and rearing modes strongly influence viral diversity in mammals [35], affecting host population dynamics and interspecies interactions. In this study, we documented the habitat characteristics of the Bactrian camel sample location. The results of the α-diversity analyses revealed that the Mountainous areas presented greater viral species richness and diversity than did the plains and plateaus. This can be attributed to two primary factors. First, the diverse topography and heterogeneous environment of Mountainous areas, characterized by significant variations in temperature, humidity, and vegetation coverage, likely co-creates unique viral niches. This environmental heterogeneity not only provides more opportunities for viral mutation but also may enhance the environment’s viral reservoir capacity, thereby collectively promoting increased viral diversity and complexity in community structure. Second, the higher biodiversity in Mountainous areas can lead to a greater diversity of dietary sources and contact species for Bactrian camels, which may increase the viral species richness and diversity of Bactrian camels. This hypothesis is supported by the findings of Alvanou et al., who reported that increased biodiversity influences the general microbiome in ruminants [36]. Additionally, free-ranging individuals had a significantly greater viral composition during the rearing mode, which probably increased the frequency of contact between Bactrian camels and other animals or humans, thereby increasing the probability of viral transmission and consequently increasing viral richness. This explains the predominance of bovine-, ovine-, and porcine-origin viruses in our metagenomic dataset, suggesting that cross-species transmission has occurred. Our study demonstrated that the Bc-Virome included *Papillomaviridae*, *Polyomaviridae*, CRESS DNA viruses, *Astroviridae*, *Paramyxoviridae*, *Coronaviridae*, *Tobaniviridae* and other unclassified viruses. Viral families from Xinjiang and Qinghai presented greater RNA viral diversity. Pan et al. performed a metagenomic analysis and obtained mainly CRESS-DNA viruses, Alphanodavirus, Pepper mild mottle virus, and Picornavirus. A comparison of our results with those of Pan et al. revealed a consistent predominance of DNA viruses over RNA viruses in both studies, with CRESS DNA viruses constituting the major fraction [24]. However, our study revealed a greater number and diversity of known viruses and new viruses. The observed divergence in viral communities suggests that habitat heterogeneity and sample size collectively shape the virome composition of Bactrian camels.

A total of 10 new viruses were discovered in this study: *Picornaviridae* (*n* = 1), *Adenoviridae* (*n* = 2), *Orthoherpesviridae* (*n* = 1), and *Polyomaviridae* (*n* = 6). Picornaviruses include many pathogenic viruses, and one picornavirus was identified from a Bactrian camel, forming a clade with Yunnan shrew picornavirus 1 from Anoursorex and sharing less than 45% amino acid sequence identity with all known picornaviruses. However, their epidemiology and pathogenicity have not been characterized [37]. Therefore, this rapidly evolving and easily transmitted virus requires continued attention to prevent the emergence of disease-causing variants [38]. We also identified two types of adenoviruses from Bactrian camels: adenoviruses that infect almost all known vertebrates and fowl adenoviruses (FAdVs) [39]. However, the mechanism underlying their pathogenicity in Bactrian camels requires further investigation. Herpesviruses are discovered in a wide range of vertebrates and are highly adapted to their hosts, with severe infection usually observed only in fetuses, very young, immunocompromised, or alternative hosts [40]. MEEQ-Dpol-1283 revealed the presence of potentially novel or highly divergent herpesviruses, which cluster with *Gammaherpesvirinae* in a major clade but form a distinct subclade, sharing 60.73–65.00% amino acid identity. Finally, PyVs are small viruses with circular dsDNA genomes, some of which are associated with cancer in human or animal hosts. Information on the zoonotic potential of PyVs in mammals is extremely limited. We identified six novel polyomaviruses from Bactrian camels, three of which clustered within the human polyomavirus clade, whereas the other three formed a separate clade with bovine-derived and ovine-derived polyomaviruses. This finding suggests a potentially greater risk of cross-species transmission to humans.

We found at least 12 viruses with zoonotic potential from Bactrian camels, including MRV1, PIV5, BcoV, HEV, porcine astrovirus, bovine torovirus and goat torovirus, and HPV et al. Notably, these viruses appeared to be non-pathogenic in Bactrian camels, yet they are linked to various diseases in humans or other animals, suggesting that Bactrian camels may act as asymptomatic reservoirs for these pathogens.

Viral cross-species transmission is the primary driver behind the increase in the incidence of emerging infectious diseases [20,41]. A thorough understanding of virus–host interactions is crucial for predicting future emergence events, particularly for viruses with pathogenic potential in humans [42]. In this study, we identified BcoV, PAstV, MRV 1, and HPV from Bactrian camels. These sequences presented >90% nucleotide sequence identity, indicating possible transmission from these hosts to Bactrian camels. Although our analysis cannot establish definitive evidence regarding transmissibility potential, these findings can help select high-priority viruses to conduct further studies on their possible emergence threats in animal and human populations.

We identified multiple pathogenic viruses with zoonotic potential that affect both animals and humans, including HEV-8 and PIV5. HEV-8 represents one of the genotypes within the genus *Paslahepevirus* and is found only in Bactrian camels. The confirmed zoonotic transmission and pathogenicity of HEV1-7 in human and animal populations suggest possible undiscovered risks from HEV-8, although no human infections have been reported. Parainfluenza virus (PIV) is an important human and animal pathogen of the central nervous system and the respiratory system [43]. PIV5 circulates among humans and animals, suggesting that it may pose a challenge to the health of both humans and animals. Therefore, continuous surveillance needs to be performed to generate information that can help prevent and control outbreaks of these viruses in the future. Our analysis was based on sequences of small amplicons. To validate these findings, viral isolation and cultivation should be performed to confirm their existence and characterize their biological properties. Additionally, HEV-8 and PIV5 were identified in samples from Aksu (Xinjiang) and Jinchang (Gansu), respectively. Notably, the sampling locations are characterized by distinct environmental and husbandry practices: Aksu is a mountainous area with captive farming, whereas Jinchang is a plain area with free-ranging practices. Both locations are associated with either mountainous areas or free-ranging, which were previously linked to increased viral diversity. These conditions may not only influence viral ecology but also potentially facilitate the transmission of pathogenic viruses.

In addition to the aforementioned mammalian viruses, several plant-associated viruses from Bactrian camels have also been identified, including members of the families *Alphaflexiviridae*, *Betaflexiviridae*, *Solemoviridae*, *Tombusviridae* and *Virgaviridae*. Some studies have also reported cases of plant viruses being carried by mammals [44,45]. As herbivores, Bactrian camels may acquire these plant-derived viruses through the ingestion of contaminated food and water [46]. The natural presence of plant viruses (such as the members of *Alphaflexiviridae*, *Virgariridae* and *Tombusviridae*) in mammals has been reported in several studies, and these mammals may spread infectious phytoviruses that are stable in the gastrointestinal tract [47]. Furthermore, the reports indicate that some plant-derived viruses can affect the intestinal bacterial composition and suggest the possibility of a direct or indirect pathogenic role for the mammal host [48,49]. Therefore, our findings suggest that Bactrian camels may act as effective disseminators for plant viruses. The mobility of Bactrian camels could facilitate the broad dispersal of these viruses across the landscape. Notably, as some of the most abundant viruses detected across sampling locations, these plant viruses might influence the gut microbiota composition of Bactrian camels. The long-term presence of these bacteria could potentially impact host health, although we found no direct evidence linking them to overt disease. Consequently, the transmission dynamics, pathogenic and ecological role of plant viruses in Bactrian camels warrant further investigation.

This study had several limitations. First, considering the large population of nasal, pharyngeal, and anal swab samples, we pooled all the swab samples from the same sampling location to construct a DNA and RNA library. The sample pooling strategy could be strongly influenced by the small number of swab samples that carry abundant viruses, which would prevent us from determining prevalence rates, coinfection rates, individual-level risk factors, and limit the ecological findings. Therefore, RT-PCR/PCR would be used to assess the viral distribution, coinfection rates and pathogenesis of specific viruses among individual camel. Additionally, nasopharyngeal swabs from the respiratory system and anal swabs from the digestive system will be pooled separately to help ascertain the virus’s predilection site and pathogenesis in our subsequent research. Furthermore, building upon these metagenomic findings, targeted single-virus genomics or single-cell sequencing may provide deeper insights into Bc-Virome [50]. Second, given the vast population size of Chinese Bactrian camels, the current sample size is limited, potentially affecting the comprehensive characterization of viral composition and diversity [51,52,53]. Studies with a larger sample size are needed to understand the viral diversity in Bactrian camels more comprehensively. These results revealed that Bactrian camels play an important role as viral hosts, necessitating the development of robust public health monitoring and prevention strategies.

In summary, this study provides valuable data for a better understanding of the existing viral population and diversity within Bactrian camel populations in China. We discovered 10 novel DNA and RNA viruses belonging to three families. Putative cross-species transmissions were observed with some identified viruses. The baseline virome profile of Chinese Bactrian camels was constructed in this study. Our findings lay the foundation for further studies on a wider range of viral lineages before potential spillover events into human populations.

## 5. Conclusions

This study delves into viral diversity and composition in Chinese Bactrian camels, which are not only vital for the food supply but also play a pivotal role in the prevention and control of critical viral diseases. Focusing on Bactrian camels, we obtained 3262 viral contigs, which were classified into 16 viral phyla, 29 viral families, and an unclassified group, highlighting the vast viral diversity within Bactrian camels. Concurrently, the Mountainous areas demonstrated greater viral diversity and composition. We identified at least 12 viruses with zoonotic potential from Bactrian camels. Phylogenetic analysis indicated that some of them may exhibit cross-species transmission. This finding suggests that viruses may move between different mammals more frequently than previously thought. Picornavirus, polyomavirus, and CRESS DNA viruses from Bactrian camels may represent novel species or genotypes. This knowledge is crucial for describing the baseline virome profile of Chinese Bactrian camels and managing disease risk in livestock production.

## Data Availability

The raw data from the meta-transcriptomic sequencing generated in this study are available at the NCBI Sequence Read Archive (SRA) database under the BioProject accession PRJNA1284745 and PRJNA1287231. The Biosample numbers were SRR34397655~SRR34397676, SRR34396925~SRR34396946. The viral sequences generated in this study have been deposited in GenBank. The serial numbers were PX096857-PX096874, PX113364-PX113376, PX113227-PX113295.

## Supplementary Materials

The following supporting information can be downloaded at: https://www.mdpi.com/article/10.3390/microorganisms13112589/s1, Table S1: Bactrian Camel Sample Collection Information Form; Table S2: Primers and Positivity Rates for Detection of Selected Viruses in Bactrian Camels; Table S3: Metagenomic sequencing data of swab samples from Bactrian camel; Table S4: title The counts of Sample Pool and Animal by Different Breeding Mode, Age, and Landform; Figure S1: Composition and diversity of Bc-Virome.
