# Supplementary material for: Metagenomic Sequencing Reveals the Viral Diversity of Bactrian Camels in China
Source: Microorganisms. 2025 Nov 13;13(11):2589. doi: 10.3390/microorganisms13112589 (PMC12654277; doi:10.3390/microorganisms13112589)
Supplement: Supplementary file 1 [file microorganisms-13-02589-s001.zip › Table S4 The counts of Sample Pool and Animal by Different Breeding Mode, Age, and Landform.docx]

### **Table S4 The Counts of Sample Pool and Animal by Different Breeding Mode, Age, and Landform**

| **Samples**  **Categories** | | **Sampling pools** | | **Bactrian Camels** | |
| --- | --- | --- | --- | --- | --- |
|  |  | **Number** | **Percent (%)** | **Number** | **Percent (%)** |
| **Landforms** | **Plateau** | 4 | 18.18 | 142 | 20.26 |
|  | **Mountain** | 5 | 22.73 | 151 | 21.54 |
|  | **Plain** | 13 | 59.09 | 408 | 58.20 |
| **Rearing modes** | **Free-ranging** | 11 | 50 | 327 | 46.65 |
|  | **Captive** | 11 | 50 | 374 | 53.35 |
| **Ages** | **Infancy** | 8 | 36.36 | 265 | 37.80 |
|  | **Juvenile** | 6 | 27.28 | 154 | 21.97 |
|  | **Middleage** | 8 | 36.36 | 282 | 40.23 |
