# Supplementary material for: Metagenomic Sequencing Reveals the Viral Diversity of Bactrian Camels in China
Source: Microorganisms. 2025 Nov 13;13(11):2589. doi: 10.3390/microorganisms13112589 (PMC12654277; doi:10.3390/microorganisms13112589)
Supplement: Supplementary file 1 [file microorganisms-13-02589-s001.zip › Supplement Figure 1. Composition and diversity of BC-Virome.docx]

**Supplementary material**


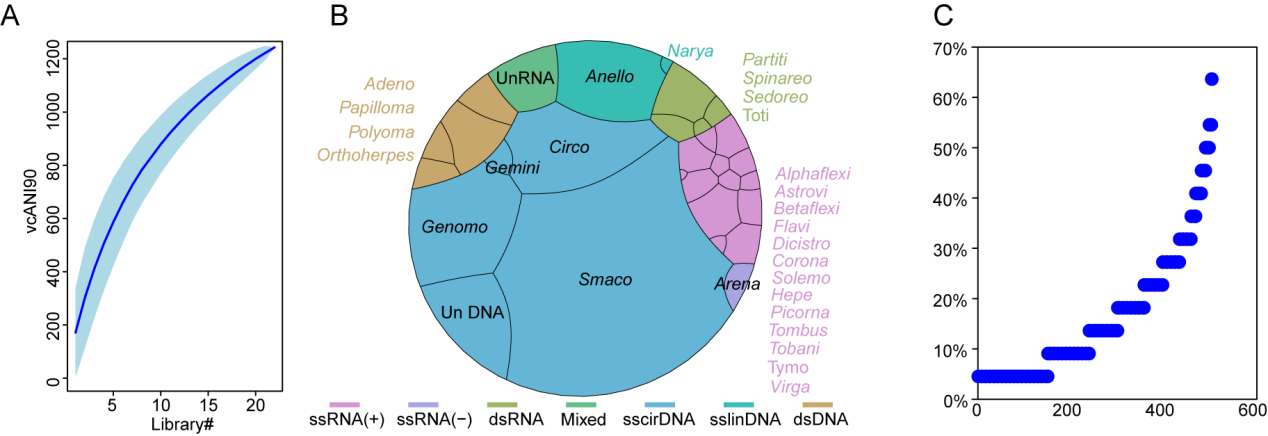


**Supplement Figure 1. Viral composition and diversity of BC-Virome.** A: The vcAAI90 accumulation curves for different samples are illustrated. The X-axis represents the number of viral reads obtained from the library, and the Y-axis represents the number of viral species observed in the Bc-Virome; B: Viral genomic structural types of the Bc-Virome; The color gradient is used to represent distinct viral genomic structural types, and the areas in the chart are proportional to the quantity of each type; C: The relative proportions of different viral species in the sequencing library. Each point denotes a vcAAI90 (viral cluster at 90% amino acid identity), indicating its detection frequency among all libraries.
