# Supplementary material for: Metagenomic Sequencing Reveals the Viral Diversity of Bactrian Camels in China
Source: Microorganisms. 2025 Nov 13;13(11):2589. doi: 10.3390/microorganisms13112589 (PMC12654277; doi:10.3390/microorganisms13112589)
Supplement: Supplementary file 1 [file microorganisms-13-02589-s001.zip › Table S1 Bactrian Camel Sample Collection Information Form.docx]

**Table S1 Bactrian Camel Samples Collection Information Form**

| **Number** | **Sample ID** | **Collection Location** | **Collection Date** | **Sample Type** | **Herd size** | **Sample size** | **Landforms** | **Rearing modes** | **Ages** |
| --- | --- | --- | --- | --- | --- | --- | --- | --- | --- |
| 1 | MAZQ | Inner Mongolia: Alxa Left Banner | 2023.4 | Nasal, pharyngeal, and anal Swabs | 200 | 31 | Plateau | Free-ranging | Middleage |
| 2 | MAEN | Inner Mongolia: Ejin Banner | 2023.4 | Nasal, pharyngeal, and anal Swabs | 160 | 31 | Mountainous area | Free-ranging | Infancy |
| 3 | MAYQ | Inner Mongolia: Alxa Right Banner | 2023.4 | Nasal, pharyngeal, and anal Swabs | 210 | 30 | Mountainous area | Free-ranging | Middleage |
| 4 | MBWH | Inner Mongolia: Bayan Nur | 20.23.3 | Nasal, pharyngeal, and anal Swabs | 1200 | 34 | Plain | Captive | Middleage |
| 5 | MXSZ | Inner Mongolia: Xilingol League | 2023.5 | Nasal, pharyngeal, and anal Swabs | 363 | 40 | Plain | Captive | Middleage |
| 6 | MWSZ | Inner Mongolia:Ulanqab | 2023.5 | Nasal, pharyngeal, and anal Swabs | 790 | 61 | Plain | Captive | Middleage |
| 7 | MTDM | Inner Mongolia:Baotou | 2023.5 | Nasal, pharyngeal, and anal Swabs | 78 | 30 | Mountainous area | Free-ranging | Juvenile |
| 8 | MEEQ | Inner Mongolia:Ordos | 2023.2 | Nasal, pharyngeal, and anal Swabs | 40 | 20 | Plain | Free-ranging | Juvenile |
| 9 | GWMQ | Gansu:Wuwei | 2023.3 | Nasal, pharyngeal, and anal Swabs | 450 | 30 | Mountainous | Free-ranging | Juvenile |
| 10 | GCSW | Gansu:Jinchang | 2023.4 | Nasal, pharyngeal, and anal Swabs | 280 | 30 | Plain | Free-ranging | Infancy |
| 11 | GZPH | Gansu:Zhangye | 2023.4 | Nasal, pharyngeal, and anal Swabs | 220 | 31 | Plain | Captive | Middleage |
| 12 | GJSB | Gansu:Subei | 2023.4 | Nasal, pharyngeal, and anal Swabs | 80 | 31 | Plateau | Captive | Infancy |
| 13 | GJGZ | Gansu:Guazhou | 2023.4 | Nasal, pharyngeal, and anal Swabs | 180 | 20 | Plain | Free-ranging | Juvenile |
| 14 | QXDL | Qinhai:Ordos | 2023.7 | Nasal, pharyngeal, and anal Swabs | 350 | 36 | Plateau | Free-ranging | Infancy |
| 15 | QXWM | Qinhai:Haixi | 2023.6 | Nasal, pharyngeal, and anal Swabs | 800 | 44 | Plateau | Free-ranging | Infancy |
| 16 | XCAA | Xinjiang:Changji | 2024.5 | Nasal, pharyngeal, and anal Swabs | 85 | 25 | Plain | Free-ranging | Middleage |
| 17 | XTGH | Xinjiang:Turpan | 2024.5 | Nasal, pharyngeal, and anal Swabs | 120 | 32 | Plain | Captive | Infancy |
| 18 | XWDS | Xinjiang:Dabancheng | 2024.5 | Nasal, pharyngeal, and anal Swabs | 110 | 30 | Plain | Captive | Infancy |
| 19 | XWWT | Xinjiang:Ürümqi | 2024.5 | Nasal, pharyngeal, and anal Swabs | 150 | 31 | Plain | Captive | Infancy |
| 20 | XAKP | Xinjiang:Aksu | 2024.6 | Nasal, pharyngeal, and anal Swabs | 256 | 30 | Mountainous area | Captive | Middleage |
| 21 | NSSH | Ningxia:Shizuishan | 2024.11 | Nasal, pharyngeal, and anal Swabs | 210 | 25 | Plain | Captive | Juvenile |
| 22 | NZSP | Ningxia:Zhongwei | 2024.11 | Nasal, pharyngeal, and anal Swabs | 121 | 29 | Plain | Captive | Juvenile |
