# Supplementary material for: Metagenomic Sequencing Reveals the Viral Diversity of Bactrian Camels in China
Source: Microorganisms. 2025 Nov 13;13(11):2589. doi: 10.3390/microorganisms13112589 (PMC12654277; doi:10.3390/microorganisms13112589)
Supplement: Supplementary file 1 [file microorganisms-13-02589-s001.zip › Table S2 Primers and Positivity Rates for Detection of Selected Viruses in Bactrian Camels.docx]

**Table S2 Primers and Positive Rates for Detection of Selected Viruses in Bactrian Camels**

| **Virus** | **Primer Name** | **Primers(5’-3’)** | **Product Size** | **PCR Positive Rate** |
| --- | --- | --- | --- | --- |
| Bovine coronavirus | BcoV-OF1 | AAGGTGTGCCTATTGCACCA | 444 | 3.7% (3/81) |
|  | BcoV-OR1 | TGACGCTGTGGTTTTGGACT |  |  |
|  | BcoV-F1 | GACCAGTATGGCACCGACAT |  |  |
|  | BcoV-R1 | GGGCCTCTCTTCCCAAAACA |  |  |
| Paslahepevirus | HEV-OF1 | TGATTTAGTTGCCCGTGCCT | 296 | 16.3% (15/92) |
|  | HEV-OR1 | AAAGAGTGCTCTCGCCACAA |  |  |
|  | HEV-F1 | TACAGCAGGTTCCAGTCAGC |  |  |
|  | HEV-R1 | CAGGAGGGAAAACCACCACA |  |  |
| Porcine astrovirus | AstV-ORF1b-WR | TGGGAGTTTCTTGATGACGTCTA | 467 | 10.7% (5/75) |
|  | AstV-ORF1b-WF | CTRTTACGCCGATTGGCCAT |  |  |
|  | AstV-ORF1b-NR | TGACCGTTTAGTCTCGGTTGT |  |  |
|  | AstV-ORF1b-NF | GAMCTCCGYYGSGARAAGRTG |  |  |
| Circovirus | Cirv-F4 | CACCTTTGGTTTCTGCGGTG | 441bp | 20.3% (13/64) |
|  | Cirv-R4 | GAGGAGGTGAAGCGACCAAA |  |  |
| Bovine coronavirus-S | Bcov-SF1 | GGCTGCATGATGCTTAGACCAT | 1353bp | - |
|  | Bcov-SR1 | GCTAACAGAAACATTAGCAGC |  |  |
|  | Bcov-SF2 | CAGGCAGACTCATTTACTTGT | 961bp |  |
|  | Bcov-SR2 | CGACCACTATAGCAACTACG |  |  |
|  | Bcov-SF3 | CACAGACATAATTCTTGGTGTTTG | 1280bp |  |
|  | Bcov-SR3 | GCATTTGCATTAACAACAGCTTG |  |  |
|  | Bcov-SF4 | AGTGGCACCAGACTTGTCAC | 460bp |  |
|  | Bcov-SR4 | ACCCACTAAACAGCAGGCAT |  |  |
