# Supplementary material for: Metagenomic Sequencing Reveals the Viral Diversity of Bactrian Camels in China
Source: Microorganisms. 2025 Nov 13;13(11):2589. doi: 10.3390/microorganisms13112589 (PMC12654277; doi:10.3390/microorganisms13112589)
Supplement: Supplementary file 1 [file microorganisms-13-02589-s001.zip › Table S3 Metagenomic sequencing data of swab samples from Bactrian camel.docx]

**Table S3 Metagenomic Sequencing Data of Swab Samples from Bactrian Camels**

| **Number** | **Sample** | **Raw Reads** | **Clean Reads** | **Raw Base(G)** | **Clean Base(G)** | **Effective(%)** | **Error(%)** | **Q20(%)** | **Q30(%)** | **GC(%)** |
| --- | --- | --- | --- | --- | --- | --- | --- | --- | --- | --- |
| 1 | MAZQ-RNA | 69614628 | 63505082 | 10 | 10 | 91 | 0 | 97 | 93 | 45 |
| 2 | MAEN-RNA | 66356342 | 62740518 | 10 | 9 | 95 | 0 | 98 | 94 | 48 |
| 3 | MAYQ-RNA | 67154456 | 65865980 | 10 | 10 | 98 | 0 | 98 | 94 | 48 |
| 4 | MBWH-RNA | 68984946 | 67065606 | 10 | 10 | 97 | 0 | 98 | 94 | 49 |
| 5 | MXSZ-RNA | 142156558 | 139524258 | 21 | 21 | 98 | 0 | 98 | 93 | 53 |
| 6 | MWSZ-RNA | 45250936 | 45164108 | 7 | 7 | 100 | 0 | 98 | 93 | 39 |
| 7 | MTDM-RNA | 69114726 | 68301278 | 10 | 10 | 99 | 0 | 98 | 95 | 49 |
| 8 | MEEQ-RNA | 68662558 | 66176280 | 10 | 10 | 96 | 0 | 98 | 94 | 45 |
| 9 | GWMQ-RNA | 78260270 | 73925972 | 12 | 11 | 94 | 0 | 96 | 90 | 56 |
| 10 | GCSW-RNA | 142956924 | 141118468 | 21 | 21 | 99 | 0 | 98 | 93 | 43 |
| 11 | GZPH-RNA | 44465454 | 44396296 | 7 | 7 | 100 | 0 | 98 | 94 | 37 |
| 12 | GJSB-RNA | 69189240 | 67994204 | 10 | 10 | 98 | 0 | 98 | 94 | 46 |
| 13 | GJGZ-RNA | 68001198 | 65425424 | 10 | 10 | 96 | 0 | 98 | 94 | 49 |
| 14 | QXDL-RNA | 68442774 | 65908712 | 10 | 10 | 96 | 0 | 97 | 93 | 48 |
| 15 | QXWM-RNA | 68656110 | 65963764 | 10 | 10 | 96 | 0 | 97 | 93 | 46 |
| 16 | XCAA-RNA | 42447340 | 40363730 | 6 | 6 | 95 | 0 | 97 | 92 | 52 |
| 17 | XTGH-RNA | 46189056 | 44859080 | 7 | 7 | 97 | 0 | 98 | 96 | 52 |
| 18 | XWDS-RNA | 45180400 | 43603422 | 7 | 7 | 97 | 0 | 98 | 95 | 51 |
| 19 | XWWT-RNA | 42217216 | 40640290 | 6 | 6 | 96 | 0 | 98 | 95 | 52 |
| 20 | XAKP-RNA | 50605046 | 48462090 | 8 | 7 | 96 | 0 | 98 | 94 | 53 |
| 21 | NSSH-RNA | 47360926 | 46563218 | 7 | 7 | 98 | 0 | 98 | 96 | 53 |
| 22 | NZSP-RNA | 41235138 | 40525856 | 6 | 6 | 98 | 0 | 99 | 96 | 53 |
| 23 | MAZQ-DNA | 69019538 | 68747334 | 10.35 | 10.31 | 99.61 | 0.03 | 97.09 | 91.98 | 40.49 |
| 24 | MAEN-DNA | 70011752 | 69602426 | 10.5 | 10.44 | 99.42 | 0.03 | 96.65 | 91.38 | 41.1 |
| 25 | MAYQ-DNA | 66395424 | 66189616 | 9.96 | 9.93 | 99.69 | 0.03 | 97.41 | 92.65 | 41.26 |
| 26 | MBWH-DNA | 68322138 | 68020772 | 10.25 | 10.2 | 99.56 | 0.03 | 97.29 | 92.41 | 40.62 |
| 27 | MXSZ-DNA | 72387692 | 71099522 | 10.86 | 10.66 | 98.22 | 0.03 | 97.69 | 93.32 | 40.84 |
| 28 | MWSZ-DNA | 71740240 | 71383440 | 10.76 | 10.71 | 99.5 | 0.03 | 97.51 | 92.93 | 43.36 |
| 29 | MTDM-DNA | 69238070 | 68984128 | 10.39 | 10.35 | 99.63 | 0.03 | 97.37 | 92.59 | 39.92 |
| 30 | MEEQ-DNA | 67657066 | 67449368 | 10.15 | 10.12 | 99.69 | 0.03 | 97.48 | 92.8 | 38.23 |
| 31 | GWMQ-DNA | 45445584 | 45320694 | 6.82 | 6.8 | 99.73 | 0.03 | 97.84 | 93.77 | 40.04 |
| 32 | GCSW-DNA | 81687842 | 80656406 | 12.25 | 12.1 | 98.74 | 0.03 | 97.94 | 93.86 | 39.37 |
| 33 | GZPH-DNA | 44465454 | 44396296 | 6.67 | 6.66 | 99.84 | 0.03 | 97.92 | 93.75 | 37.48 |
| 34 | GJSB-DNA | 70130850 | 69559446 | 10.52 | 10.43 | 99.19 | 0.03 | 97.18 | 92.26 | 39.05 |
| 35 | GJGZ-DNA | 67968708 | 67801082 | 10.2 | 10.17 | 99.75 | 0.03 | 97.22 | 92.08 | 38.98 |
| 36 | QXDL-DNA | 45888836 | 45795676 | 6.88 | 6.87 | 99.8 | 0.03 | 97.82 | 93.65 | 40.6 |
| 37 | QXWM-DNA | 44824990 | 44710942 | 6.72 | 6.71 | 99.75 | 0.03 | 97.67 | 93.19 | 37.4 |
| 38 | XCAA-DNA | 45147882 | 44752622 | 6.77 | 6.71 | 99.12 | 0.01 | 98.81 | 96.41 | 41.54 |
| 39 | XTGH-DNA | 46365104 | 45891924 | 6.95 | 6.88 | 98.98 | 0.01 | 98.86 | 96.58 | 41.27 |
| 40 | XWDS-DNA | 45284816 | 44861784 | 6.79 | 6.73 | 99.07 | 0.01 | 98.7 | 96.14 | 43.57 |
| 41 | XWWT-DNA | 44600942 | 44228172 | 6.69 | 6.63 | 99.16 | 0.01 | 98.96 | 96.93 | 43.06 |
| 42 | XAKP-DNA | 53495304 | 52854580 | 8.02 | 7.93 | 98.8 | 0.01 | 98.83 | 96.73 | 42.64 |
| 43 | NSSH-DNA | 67266226 | 66903258 | 10.09 | 10.04 | 99.46 | 0.01 | 99.26 | 97.69 | 39.95 |
| 44 | NZSP-DNA | 62257604 | 61800272 | 9.34 | 9.27 | 99.27 | 0.01 | 99.19 | 97.51 | 42.85 |
